# Smart bactericidal textile enabling in-situ visual assessment of antimicrobial activity

**DOI:** 10.1016/j.mtbio.2025.101724

**Published:** 2025-04-03

**Authors:** Amparo Ferrer-Vilanova, Josune Jimenez Ezenarro, Kristina Ivanova, Óscar Calvo, Ilana Perelshtein, Giulio Gorni, Ana Cristina Reguera, Rosalía Rodríguez-Rodríguez, Maria Blanes, Núria Vigués, Jordi Mas, Aharon Gedanken, Tzanko Tzanov, Gonzalo Guirado, Xavier Muñoz-Berbel

**Affiliations:** a Institut de Microelectrònica de Barcelona (IMB-CNM, CSIC), Universitat Autònoma de Barcelona, 08193, Cerdanyola del Vallès, Barcelona, Spain; b Universitat Politècnica de Catalunya, Edifici Gaia, Pg. Ernest Lluch/Rambla Sant Nebridi s/n., 08222, Terrassa, Barcelona, Spain; c Asociación de Investigación de la Industria Textil – AITEX, Área de I+D, Grupo de Investigación en Eco-procesos, Cosmética y Salud. Plaza Emilio Sala, 1, 03801, Alcoi, Alacant, Spain; d Department of Chemistry, and the BINA center, Bar-Ilan University, 5290002, Ramat-Gan, Israel; e CELLS-ALBA Synchrotron, Carrer de la Llum 2-26, 08290, Cerdanyola del Vallès, Barcelona, Spain; f Instituto de Óptica (IO-CSIC), c/Serrano 121, 28006, Madrid, Spain; g Department of Biomedicine, Faculty of Medicine and Health Sciences, Universitat Internacional de Catalunya (UIC), Sant Cugat del Vallès, E-08195, Spain; h Centro de Investigación Biomédica en Red de Fisiopatología de la Obesidad y la Nutrición (CIBEROBN), Instituto de Salud Carlos III, Madrid, E-28029, Spain; i Departament de Genètica i Microbiologia, Universitat Autonòma de Barcelona, 08193, Cerdanyola del Vallès, Barcelona, Spain; j Departament de Química, Universitat Autonòma de Barcelona, 08193, Cerdanyola del Vallès, Barcelona, Spain; k CIBER de Bioingeniería, Biomateriales y Nanomedicina, Instituto de Salud Carlos III, Madrid, E-28029, Spain

**Keywords:** Smart textiles, Bacterial sensing, Metabolic indicators, Sonochemical coating, Antibacterial material, Nosocomial infections

## Abstract

Hospital fabrics and wound dressings with antibacterial properties are essential to minimize infection risks associated with bacterial colonization of textiles. A key challenge of these materials lies in the difficulty in assessing their functional lifespan. Integrating bacterial-sensing elements into smart textiles enables real-time and in-situ evaluation of antibacterial activity. However, this approach is often hindered by the reactivity between bactericidal and bacterial-sensing components, the limited stability and selectivity of the sensing probes, and high production costs. Here, we address these challenges by presenting a smart textile that simultaneously provides antibacterial activity and bacterial-sensing capacity using a layer-by-layer sonochemical deposition method. Prussian blue, a chromogenic bacterial-sensing probe, is integrated onto hospital-grade antibacterial fabrics containing copper oxide nanoparticles. When the biocidal fabric begins to lose its antimicrobial activity, live bacteria in the textile metabolically reduce Prussian blue nanoparticles, triggering a visible colour change. This approach offers several key advantages, such as: (i) the resulting textile retains antibacterial activity comparable to conventional copper oxide-based textiles (A value > 4 in both cases); (ii) it provides a direct and visible colour transition from blue to colourless (>20 % colour losses) when the antibacterial coating begins to lose effectiveness, enabling straightforward monitoring of antibacterial lifespan without external instruments or reagents; (iii) the co-immobilization enhances coating stability, nearly doubling the binding strength of copper oxide and Prussian blue compared to single-layer coatings; and (iv), the additional Prussian blue layer significantly reduces the material cytotoxicity, enhancing biocompatibility for safer use in healthcare settings. These innovations offer a scalable, cost-effective, and multifunctional solution for infection control. The smart textile not only prevents bacterial spread but also provides timely, visual indications of coating degradation, making it a promising tool for improving patient safety in hospitals and for minimizing infection risks in schools and other high-risk environments.

## Highlights

• Smart biocide textile enables in-situ, real-time visual assessment of antibacterial activity.
• Prussian blue acts as a bacterial-sensing probe, changing colour upon coating degradation.
• Sonochemical co-deposition of CuO and Prussian blue enhances CuO stability, reducing cytotoxicity while retaining efficacy.
• Metabolic activity of live bacteria induces reversible decolouration for infection risk monitoring.
• Potential for hospital, school, and healthcare use to improve infection control.

## Graphical abstract

**Figure:**
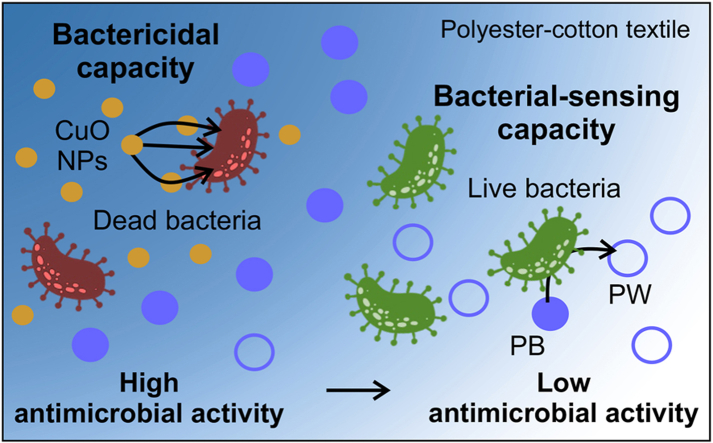

## 1 Introduction

Cotton-based fabrics are extensively used in hospitals for their comfort and breathability. They are particularly beneficial for wound dressings, as they adhere less to burns and lacerations than other materials. Additionally, their high absorbency maintains a moist environment that promotes wound healing, while preventing excessive exudate accumulation that can lead to skin maceration and bacterial proliferation [1].

Among cotton-based materials, polyester-cotton blends are preferred in hospital environments due to their enhanced durability, wrinkle resistance, and quick-drying, making them ideal for high-use medical textiles [2]. Beyond these advantages, polyester-cotton textiles also play a crucial role in infection control, as hospital fabrics can serve as pathogen reservoir, contributing to hospital-acquired infections (HAIs). In fact, the National Institutes of Health (NIH) estimates that 60–70 % of the 8.9 million HAIs reported annually in European hospitals are associated with biofilm formation [3], leading to approximately 37,000 direct deaths per year and economic losses exceeding €7 billion annually [[4], [5], [6], [7]]. In this sense, polyester-cotton fabrics reduce bacterial colonization compared to pure cotton. The rougher fibre structure of cotton provides a larger surface area for bacterial attachment, whereas the smoother polyester fibres limit bacteria adhesion. Additionally, surface wettability plays a role, as hydrophobic materials like polyester hinder bacterial attachment by reducing wetting [8]. However, despite these advantages, polyester-cotton textiles remain susceptible to bacterial colonization, necessitating effective disinfection strategies.

Currently, disinfection is the primary method used to prevent pathogen transmission from textiles, yet standard hospital laundering cannot fully eliminate bacterial contamination [9]. In wound dressings, frequent replacement and disinfection disrupts tissue healing, causing patient discomfort, prolonging recovery times, and increasing treatment costs [[10], [11], [12]]. While biocidal agents incorporated in hospital fabrics have been shown to reduce microbial colonization [[13], [14], [15]], the lack of real-time infection monitoring often leads to unnecessary dressing replacements. This highlights a critical gap in the ability to monitor bacterial activity in real-time and accurately assess infection risks.

To address this challenge, smart textiles have been developed that integrate antibacterial and bacterial-sensing functionalities. These materials detect live bacteria in the textile through colorimetric or fluorescence-based changes, providing a visual indication of potential infection. Smart bacterial-sensing materials typically employ one of four main types of detection probes: i) phospholipid membranes containing fluorescent or colorimetric molecules that are disrupted by bacterial lytic agents (e.g. toxins) [16,17]; ii) organic dyes that respond to physicochemical changes associated with bacterial metabolism, such as pH shifts [18,19]; iii) oxygen-sensitive compounds that change colour due to oxygen depletion caused by microbial respiration [[20], [21], [22]]; and iv) metabolic indicators that are directly reduced by bacterial electron transport chain proteins [23].

Although these materials share similar detection limits and response times, they differ in selectivity, stability, and scalability. Phospholipid membranes offer high selectivity for specific bacterial toxins, but they are unstable and challenging to produce at scale. Oxygen-sensitive compounds, i.e. Eu^3+^ and Methylene blue, are more stable, but Eu^3+^ is cytotoxic and both compounds lack scalability for textiles. Organic dyes can be integrated into textiles using scalable technologies [24], but they provide indirect and less selective bacterial detection, making them prone to false positives from environmental interferences.

Among metabolic indicators, Prussian blue (PB) stands out due to its potential integration into hospital fabrics via scalable technologies, such as sonochemical coating [23]. PB coatings are durable [25], specifically reduced by bacterial electron transport chain proteins [26,27], and effective against both Gram-positive and Gram-negative bacteria [23]: PB-based bacterial detection has been previously demonstrated for *Escherichia coli* (*E. coli*) [26,28], *Pseudomonas aeruginosa,* [29] *Staphylococcus aureus,* [23,30] and *Staphylococcus capitis.* [31] However, its applicability in fungal detection has not yet been established.

The co-immobilization of PB with biocidal metal or metal oxide NPs has been previously explored in silver-PB composites for antibacterial glass surfaces [32], and in Co-doped PB-modified hollow polydopamine for wound care [33]. However, this approach has not yet been investigated for textiles.

This article focuses on the co-immobilization of PB with CuO-NPs on polyester-cotton textiles using a layer-by-layer sonochemical coating process. CuO-NPs were selected for their well-documented antibacterial and antifungal properties [34], non-irritating nature, lack of sensitization, and minimal adverse effects, key characteristics for textile applications [13]. The resulting smart textiles were evaluated for their antibacterial and bacterial-sensing capabilities and compared against textiles containing either PB or CuO-NPs alone to assess potential cross-reactivity and synergistic effects between the two nanoparticulate systems. The combination of PB and CuO-NPs was specifically designed to enhance both performance and stability through a redox-based synergistic mechanism, similar to those observed in core@shell bimetallic particles [35,36]. In such system, the ability of one nanoparticulate component to restore the redox state of the other leads to enhanced antibacterial activity. Likewise, the redox properties of PB and CuO-NPs are expected to contribute to continuous regeneration of their initial redox states, thereby improving and expanding antibacterial performance while enhancing system stability.

To validate this hypothesis, *E. coli* was selected as model microorganism due to the broad-spectrum antibacterial activity of CuO-NPs and the previously demonstrated ability of PB-coated textiles to detect both Gram-positive and Gram-negative bacteria [26]. This approach not only leveraged the established biocidal effect of CuO-NPs, but also explored a novel redox-driven synergy that could be extended to other biocide/sensor combinations for the next generation of smart antimicrobial textiles.

## 2 Materials and methods

### 2.1 Reagents

Potassium ferricyanide (K_3_[Fe(CN)_6_]; ≥99 %), iron(II) chloride (FeCl_2_; 98 %), phosphate buffered saline (PBS) and 2-(N-morpholino)ethanesulfonic acid hydrate (MES hydrate; ≥99 %) were purchased from Sigma-Aldrich (Spain). Glucose, sodium borohydride (NaBH_4_; 96 %), potassium phosphate dibasic trihydrate (K_2_HPO_4_·3H_2_O; ≥99 %), potassium dihydrogen phosphate (KH_2_PO_4_; ≥99 %) and hydrogen peroxide (H_2_O_2_; 30 %) were obtained from Panreac (Spain). Soya Casein Digest Lecithin Polysorbate Broth (SCDLP) medium was acquired from Scharlab S.L. (Spain). All chemicals were used as received. Aqueous solutions were prepared using deionized water (resistivity = 17 MΩ cm).

### 2.2 Production of PB-NPs

Insoluble PB was synthetized by mixing 50 mL of 15 mM K_3_[Fe(CN)_6_] with an excess of FeCl_2_ (30 mL, 0.1 M solution). The iron dichloride solution was added carefully to the ferricyanide and a dark blue precipitate corresponding to PB-NPs was formed immediately. The precipitate was left to deposit (around 8 h) and the supernatant was removed. The pellet was resuspended in distilled water to eliminate the excess of reagents. This last step was repeated twice.

### 2.3 PB-NPs size and aggregation

Dynamic Light Scattering (DLS) was employed to determine the hydrodynamic diameter and polydispersity index (PDI) of the PB-NPs in dispersion. Measurements were performed using a Zetasizer Pro (Malvern Panalytical, UK), equipped with a 633 nm He-Ne laser at a scattering angle of 173° (backscatter detection). Samples were prepared by dispersing PB-NPs in distilled water at a concentration of 0.1 mM PB. The suspensions were sonicated for 5 min prior to analysis to minimize aggregation. Measurements were conducted at 25 °C, and data were analysed using ZS XPLORER software (Malvern Panalytical, UK).

### 2.4 Coating of polyester-cotton fabrics

Polyester-cotton fabrics were sonochemically coated with PB- and/or CuO-NPs. CuO-NPs coating was performed at Klopman Int. 10.13039/100001328SRL (Italy) using an industrial sonochemical coating machine designed under the EU-funded project PROTECT. Fabric samples (50 cm width, 5 m length) were immersed in a tank containing 0.01 M copper acetate water solution (0.2 g copper acetate for each 100 mL reaction volume). The ultrasonic generator had an output power up to 4 kW in a working range of frequencies of 20–27 kHz. The sonicator was turned on (90 % amplitude) until reaching 60 °C (5–7 min). Then, ammonia was added dropwise up to a pH 8, changing the solution colour from blue to brown due NPs formation. The rate of feed of the fabric was of 10 m/min, which 20 times faster than that reported in Ref. [37]. Homogeneous and highly coloured textile samples were obtained.

In a second step, fabric fragments (3 × 3 cm, approx. 0.18 g each) were coated with PB-NPs using an ultrasonic transducer (Ti-horn, 20 kHz, 750 W, Sonics and Materials CV334, USA) at 21.5 W and 0.43 W/cm^3^ [38]. Fabrics pieces were introduced in a pot containing 50 mL of the PB-NPs aqueous suspension and the ultrasonic tip was situated at 1 cm of the sample. The PB coating was performed under the conditions already optimized in our previous publication [23], in order to provide the samples with a homogeneous NPs distribution and intense blue colour without compromising the integrity of the textile, i.e. 0.08 mM PB-NPs, 15 min of sonication, 20 °C and 35 % amplitude.

### 2.5 SEM and EDX measurements

The surface topology of the modified textiles was studied by Field Emission Scanning Electron Microscopy (FE-SEM) using an AURIGA® series en04 SEM (CarlZeiss) coupled to Oxford Inca Energy Dispersive X-Ray Analysis (EDX) system.

The quantification of surface coverage from SEM images was performed by using the freeware *ImageJ*. The threshold function was applied to remove the image background and distinguish the particles covering the fibre on a black-and-white scale. A representative area on the fibre was then selected, and a histogram of the image was used to quantify the percentage of the area covered by the NPs.

### 2.6 XAS analysis

X-ray absorption (XAS) spectra at Fe K-edge were measured employing the CLAESS beamline at the ALBA synchrotron using a Si (311) double crystal monochromator [39]. Harmonic rejection was achieved by a proper combination of angle and coating of collimating and focusing mirrors. The beam size at the sample position was adjusted to 300 x 300 μm^2^. The beamline was calibrated using a Fe foil measured in transmission mode, with the energy of the first maximum in the derivative spectrum taken at 7112 eV. The incident and transmitted intensities were detected by two ionization chambers filled with a mixture of He/N_2_/Kr. The samples were placed at 45° to the incident beam and measured at room temperature in fluorescence mode. The fluorescence signal (Fe Kα) was detected by a six-channel silicon-drift detector. Data analysis was performed using ATHENA and ARTEMIS software [40].

### 2.7 Bacterial cultures preparation

*Escherichia coli (ATCC 25922)* (*E. coli*) was used as model microorganism for antibacterial and bacterial-sensing studies. *E. coli* was incubated aerobically in a Luria-Bertani (LB) broth overnight (18h) at 37 °C under constant shaking (Infors CH-4103 Bottingen). After centrifugation (5804 R Eppendorf centrifuge, Germany) at 2700×*g* for 10 min, the supernatant was removed and the pellet was re-suspended in 0.1 M MES (pH 6.2) or phosphate buffer (pH 7.2), adjusting the bacterial concentration through optical density measurements with a spectrophotometer (Smartspec Plus spectrophotometer, Bio-rad, California, US). Both mediums were supplemented with 0.1 % glucose. An optical density of 0.1 A U. was considered equivalent to 10^8^ colony-forming units per mL (CFU/mL) at the beginning of the experiment. Bacterial concentration was adjusted after cell culturing in agar plates and counting.

### 2.8 Antibacterial capacity

The antibacterial activity of fabric samples (test pieces with a mass of 0.40 g ± 0.05 g) was measured according to the ISO 20743:2021 procedure [41], as described in Fig. 3d. First, six samples of each type were prepared for the antibacterial test (polyester-cotton textile with PB, CuO-NPs, or both nanoparticulate systems). Non-modified polyester-cotton textiles were used as control samples. Bacterial suspensions containing 10^5^ CFU/mL (200 μL) were inoculated directly onto several points of each sample. Three of the six samples were used for zero-time determination. In zero-time determination, samples were homogenized immediately after inoculation in a stomacher with 20 mL of SCDLP. The samples and the solution were placed in stomacher bags and sealed. They were then shacked in the stomacher at 260 rpm for 1 min on each side of the bag. The extract was then inoculated on agar plates and incubated at 37 °C overnight to determine bacterial concentration by the colony counting method. The other three samples were incubated for 18–24 h at 37 °C with bacteria, then homogenized and counted as described above. Results were compared with the antibacterial activity of the control fabric according to the following formula (1):

(1) $$A=F-G=\left(\log\;C_{t}-\log\;C_{0}\right)-\left(\log\;T_{t}-\log\;T_{0}\right)$$

Where A is the antibacterial activity value; F is the growth value on the control fabric; log C_t_ and log C_0_ are the average common logarithms for the number of bacteria obtained from three test samples of control fabrics after 18–24 h incubation and immediately after inoculation, respectively; G is the growth value on the antibacterial-treated sample; and log T_t_ and log T_0_ are the average common logarithms for the number of bacteria obtained from three antibacterial-treated test samples after 18–24 h of incubation and immediately after inoculation, respectively.

### 2.9 NPs release to the medium

To determine the stability of CuO-NPs and PB-NPs on the textile, the antibacterial activity of the medium was studied, accompanied by Inductively-Coupled Plasma – Optical Emission Spectrometry (ICP-OES) analysis of the medium composition. First, textile samples were submerged in 3 mL of MES medium for 24 h. After that time, the sample was diluted in 1 % (v/v) HNO_3_ and the blank was evaluated in parallel. An aliquot (1 mL) was analysed by ICS-OES to determine Cu and Fe concentrations in MES using an optical emission spectrometer (Perkin-Elmer, optima 4300DV) and a microwave digestion station (Milestone, Ultrawave). The concentration of Cu and Fe was determined in mg/L of MES. An external calibration was performed using five traceable stock solutions of 1000 ppm Fe and Cu (between 0.01 and 1 mg/L) from NIST (Inorganic Ventures, Christiansburg, VA 24073 USA). The limit of detection was determined as the minimum quantifiable concentration in the sample after applying the dilution factors, resulting in a value of 0.03 mg/L. Statistical analysis was performed using GraphPad Prism 10.0. A *t*-test was used to compare independent groups, considering differences statistically significant at p < 0.05. Simultaneously, a second aliquot of 1.5 mL of the medium was incubated with a bacterial suspension initially containing 10^8^ CFU/mL *E. coli* for 24 h, determining the final bacterial concentration after plating and colony counting. Textiles without antibacterial treatment were used as control samples. A scheme of the protocol is illustrated in Fig. 3a.

### 2.10 Sensing and antibacterial capacities of the smart textiles

To measure the sensing and antibacterial capacities of the polyester-cotton textiles, fabrics samples (1 × 1 cm, approx. 0.02 g) modified with PB-NPs, CuO-NPs or both nanoparticulates were incubated with *E. coli* suspensions at different concentrations (10^9^, 10^8^ and 10^7^ CFU/mL) in either MES medium (pH 6.2) or in phosphate buffer (pH 7.2) supplemented with 0.1 % glucose. Both mediums were selected for providing different stability to NPs (MES increase PB stability), or a more physiological environment for bacterial proliferation (phosphate buffer is more physiological). The incubation process lasted three days and was repeated three consecutive times, with the bacterial medium refreshed every 72 h, which containing *E. coli* at the same initial concentration.

The 72-h incubation period was chosen to simulate the natural progression of *E. coli* colonization and biofilm formation under real environmental conditions. During the first 12 h, bacteria begin producing extracellular polymeric substances (EPS) and forming microcolonies [42], which then develop into mature biofilm within 24–48 h. Extending the incubation to 72 h allowed for partial maturation of the biofilm, providing a more comprehensive evaluation of bacterial behaviour on the textile surface. Additionally, medium refreshment was performed to maintain a high concentration of live bacteria, preventing nutrient depletion and supporting continued biofilm development over time.

The colour change of the textile was monitored in real time with a digital microscope camera (DigiMicro 2.0 Scale). Images were acquired at regular times (5 min) and analysed using the freeware *Image J*. For image treatment, a line was drawn along the textile surface, and the mean colour intensity was obtained for each image. The three RGB channels were separated, and the red channel (i.e. 630 nm wavelength) was selected for quantification as it was the most sensitive. A larger number of images was processed using macros, keeping the same line length for all of them to ensure comparable values. The percentage of colour lost was calculated using the initial non-modified textile as a reference. Bacterial concentrations lower than 10^7^ CFU/mL were not included in this experiment, as they were completely killed by the CuO-NPs, resulting in no colour change on the textiles.

Furthermore, the number of live bacteria in the medium was determined by colony counting on agar plates. The percentage of live bacteria was calculated by considering bacterial proliferation in solution without direct contact with textile samples (100 % viability).

### 2.11 Confocal microscopy imaging

Confocal images of the smart textiles were used to determine the number of live and dead bacteria attached to the sample after incubation to evaluate their contact killing capacity. Textiles were stained with the Live/Dead BacLight Bacterial Viability Kit (Invitrogen), as detailed by the supplier: 1.5 μL of 3.34 mM SYTO9 and 1.5 μL of 20 mM propidium iodide in 1 mL of water. Samples were incubated in 100 μL of Live/Dead staining solution for 30 min and washed with PBS. Confocal images of the smart textiles were taken with a confocal microscope (Leica TCS SP5) at excitation wavelength of 470 nm and an acquisition range between 500 and 750 nm. The laser power intensity was at 30 %, the pinhole size was 95.5 μm and a detector gain of 558. Three-dimensional reconstruction was performed with the IMARIS 9.6.1 (Bitplane, Oxford Instruments, United Kingdom) software, where live bacteria (stained with SYTO9) appeared in green (emission wavelength = 630 nm) and dead bacteria (stained with propidium iodide) emitted in the red region of the visible spectrum (emission wavelength = 530 nm). The percentage of live bacteria was obtained as the average of five different areas of identical surface.

### 2.12 Cytotoxicity assays and cell proliferation evaluation on the textile

Human adult dermal fibroblasts (HDF-a) cell line (ZenBio, Durham, NC, USA) was maintained in a humidified incubator with 5 % CO_2_ at 37 °C and cultured in Dermal Fibroblast Culture Medium (ZenBio, Durham, NC, USA). The cells used in the experiments were between the 4th to 12th passages and under 80 % confluence.

For cytotoxicity assays, HDF-a cells were seeded in a density of 40000 cells/well in 24-well plates. The cytotoxicity was determined using the colorimetric MTT (3-(4,5-dimethylthiazol-2-yl)-2,5-diphenyltetrazolium bromide) assay, based on our previous publication [23]. Briefly, HDF-a cells cultured in complete Dulbecco's Modified Eagle's Medium (DMEM, supplemented with 10 % foetal bovine serum, 1 % penicillin/streptomycin solution) were exposed to textile fragments of different area (from 6 to 50 mm^2^) and incubated for 24 h. The MTT solution (5 mg/mL) was then added and incubated for 2 h (37 °C, 5 % CO_2_). After washing with PBS, the purple formazan generated by MTT metabolism of viable cells was solubilized with dimethyl sulfoxide (DMSO; Sigma Aldrich, ≥99.5 %). The optical density of each well was determined at 570 nm in a spectrophotometer reader (BioTek® Synergy HT, Vermont, USA). Cell viability was expressed as percentage in relation to non-treated cells.

For the exploration of cell growing in the fabric, HDF-a cells were seeded in a density of 40000 cells/well over a tissue strip (0.5 × 3 cm) placed in a 24-well plate. After 48 h, cells were stained with CellTracker™ Green CMFDA (Thermo Fisher Scientific, Waltham, Massachusetts, USA) following the manufacturer instructions. That is, the CellTracker™ vial was dissolved in DMSO to a final concentration of 10 mM and warmed to 37 °C. The resulting solution was added to the samples and incubated for 30 min. After incubation, the CellTracker™ solution was removed. Representative bright field and fluorescence images were acquired using an EVOS™ M5000 Imaging System (Thermo Fisher Scientific, Waltham, Massachusetts, USA) at 4x magnification.

## 3 Results and discussion

### 3.1 Smart textiles production: mechanical, structural and functional studies

**Fig. 1:**
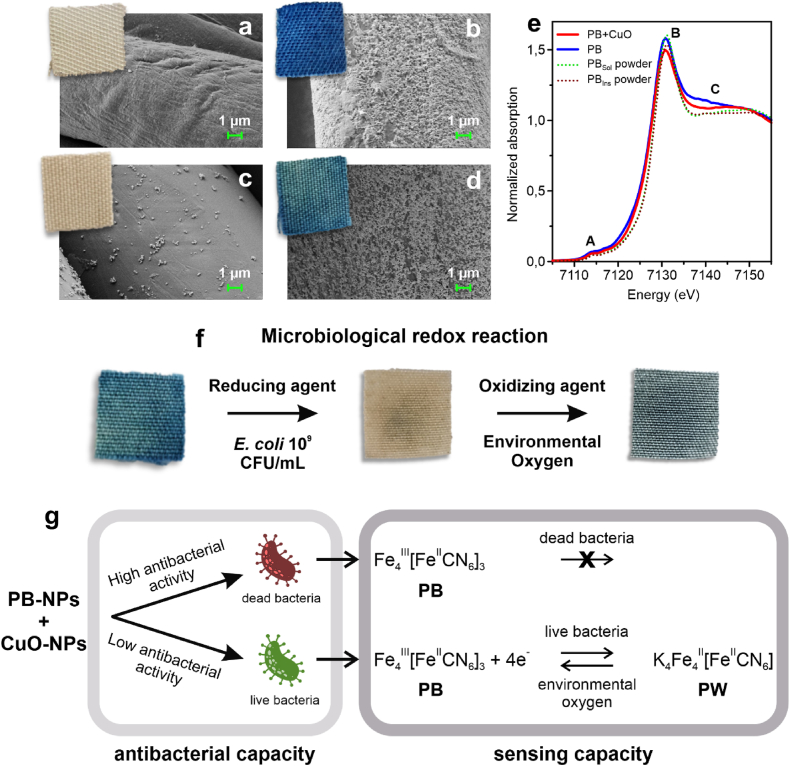
SEM images of a) polyester-cotton textile, b) polyester-cotton textile coated with PB-NPs, c) polyester-cotton textile coated with CuO-NPs, d) polyester-cotton textile coated with PB- and CuO-NPs. e) XANES spectra of soluble and insoluble PB powders and PB textile with and without CuO-NPs. f) Pictures of samples containing PB- and CuO-NPs after microbiological reduction with *E. coli* 10^9^ CFU/mL and re-oxidation by environmental oxygen. g) Schematic representation of the antibacterial and sensing performance of the smart textiles.

The cotton-polyester sample (Fig. 1a) developed a brownish colour after the first coating step (Fig. 1c), attributed to the formation of CuO-NPs, as observed by SEM. Based on a preliminary study, only samples containing ≥0.4 % copper were included in this study, as they exhibited sufficient antibacterial activity and CuO-NPs stability. Subsequent deposition of PB-NPs (hydrodynamic diameter: 76–90 nm; PDI: 6.5–7.2; details in the Supporting Information, Section S1: Dynamic Light Scattering Results) on the CuO layer resulted in a deep, homogeneous blue coloration (Fig. 1d), consistent with the formation of a dense PB coating on the textile fibres. SEM analysis confirmed an extensive PB nanoparticulate distribution, covering 92 % of the textile surface, comparable to samples coated exclusively with PB-NPs (94 % coverage) (Fig. 1b). The interaction between the coated NPs and the textile fabric is likely mediated by hydroxyl groups present in the cellulose component of the cotton fibers, as previously reported [43]. This mechanism may facilitate the binding of CuO to the textile surface serving as a nucleation site for NP formation (either CuO or PB). However, while this hypothesis aligns with existing literature, direct experimental confirmation has not yet been obtained.

The sonochemical coatings did not affect the mechanical properties of the fabric, as confirmed by tensile strength (ISO 13934–1:2013), abrasion resistance (ISO 12947–2:2016) and pilling propensity (ISO 12945–2:2000) tests, detailed in the Supporting Information (Section S2: Mechanical Properties, Table S2.1, S2.2 and S2.3). However, the coating altered the hand feel of the material, making it noticeably harder after nanoparticle deposition. Additionally, changes in the oxidative state and coordination environment of iron ions in the deposited PB-NPs were observed, as evidenced by variations in the X-ray absorption near edge structure (XANES) spectra at the Fe K-edge (around 7100 eV) when compared to soluble and insoluble PB powders.

The XANES spectra displayed three main characteristic features (Fig. 1e): (A) the pre-edge peak around 7114 eV, (B) a whiteline around 7130 eV, and (C) a multiple scattering resonance between 7140 and 7150 eV. The edge position, taken as the maximum of the first derivative spectrum, showed a 0.5 eV shift to lower energy values in the textile-bound PB-NPs than in the initial PB-NP powders, indicating a partial reduction of Fe^3+^ to Fe^2+^ during the coating. This reduction was further supported by a shift in the whiteline region from 7131.1 eV to 7130.8 eV. Additionally, deviations in the slope of feature C, attributed to the Fe^2+^–C–N–Fe^3+^ arrangement [44,45], suggested structural distortions and/or vacancies in the PB-NPs upon deposition on the textile.

Despite these structural changes, the functional response of PB-NPs remained intact. The embedded PB-NPs reacted to live bacteria, which reduced PB to colourless Prussian White (PW), causing textile discoloration (Fig. 1f). This bacterial-induced reduction required several hours and reversed upon bacterial death due to oxygen-driven re-oxidation.

Notably, textile discoloration occurs only when its antibacterial capacity declines. When the textile retains high antimicrobial activity, bacteria attaching to its surface are rapidly killed, preventing colour change. Thus, textile discoloration serves as an indicator of lost antibacterial efficacy, providing visual cue for its functional lifespan. A schematic representation of these processes is illustrated in Fig. 1g.

The presence of iron and copper in the textiles after reduction and re-oxidation steps was confirmed by EDX analysis (Supplementary Information, Section S3: EDX Measurements). Samples were immersed in a first solution containing 0.5 M NaBH_4_ as reducing agent and in a second solution with 0.5 M H_2_O_2_, as oxidizing agent, showing comparable amounts of iron and copper on the textile in both cases.

**Fig. 2:**
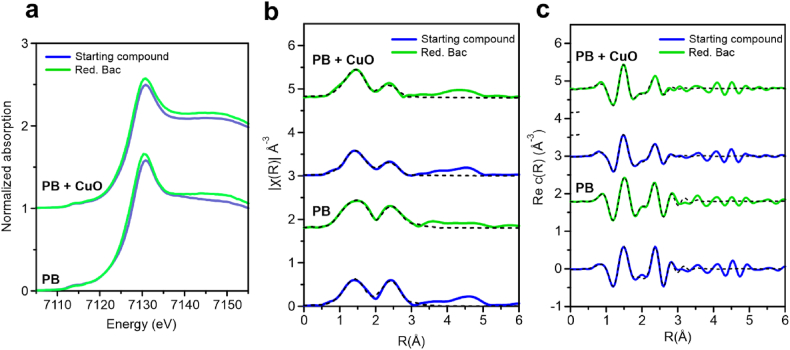
a) XANES spectra of PB textiles with and without CuO-NPs after reduction performed by bacteria. b) Fourier Transform moduli and (c) real part of k^2^*ꭓ*(k) signal obtained from EXAFS analysis. Data are represented by solid line and the fit by the dashed line.

Fig. 2a incorporates the XANES spectra of the textiles after microbiological reduction by bacteria, which was very similar in shape to those obtained by the initial PB-modified textile. Main differences between them were in the rising edge and the whiteline magnitude. Both parameters shifted by around 0.4 eV towards lower energy values, confirming the presence of slightly higher amounts of Fe^2+^ ions due to microbiological reduction. The small shift in magnitude indicated that bacteria were only reducing small amounts of Fe^3+^ to Fe^2+^, while not affecting both the local structure of PB and the shape of the whole XANES spectra. Similar conclusions were obtained for textiles also incorporating CuO-NPs and treated in the same conditions, which confirmed that the presence of the antibacterial particles did not interfere electron exchange between bacteria and PB molecules in the textile.

A deeper study of the local changes around Fe^3+^/Fe^2+^ ions after bacterial reduction process was conducted by analysing the extended X-ray absorption fine structure (EXAFS) of the previous spectra. A multiple k-weight fit was performed in the k-range 3-10 Å^−1^ and R-range 1–3.1 Å and included in Fig. 2b–c and Table S4.1 in the Supplementary Information. In previous studies, the local structure of Fe in PB was modelled considering several single scattering paths, as well as intense multiple scattering paths [[45], [46], [47]]. The first shell was related to single scattering from Fe^2+^–C and Fe^3+^–N at distances around 1.9 and 2.0 Å, respectively. Instead, the main contribution used to model the second shell was a 3-leg path around 3.0 Å due to Fe^2+^–C–N–Fe^3+^, where the linear geometry made this contribution stronger than Fe–N and Fe–C single scattering paths at similar distance. In this study, only a maximum of three paths were considered to model the data due to the short k-range available. Despite C and N may not be distinguished in EXAFS for their similar scattering properties, the shorter bond of the first shell was referred as Fe–C and the longer one as Fe–N. The same Debye-Waller factor (σ^2^) was imposed to these two scattering paths, following a procedure developed by other authors [45].

**Fig. 3:**
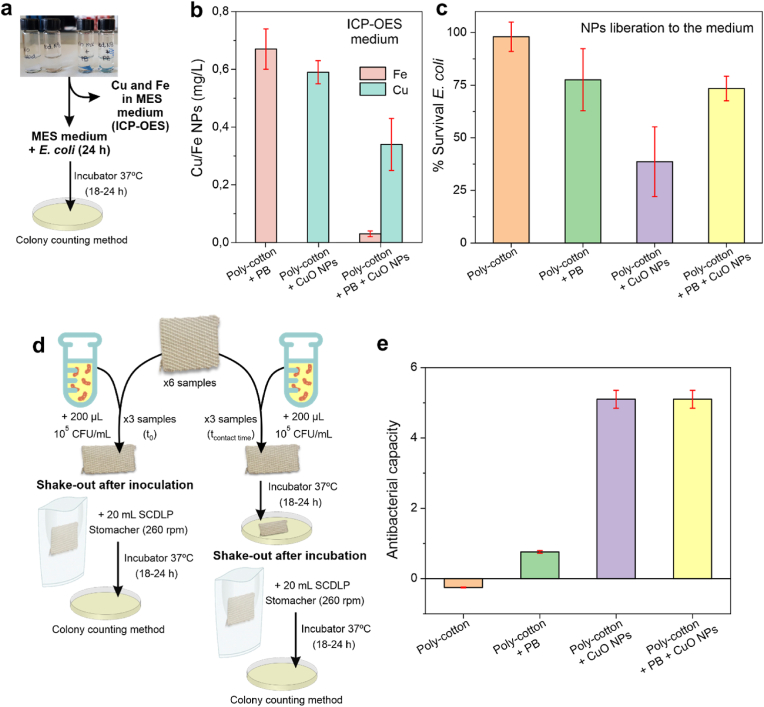
a) Scheme of the process followed for the study of Cu and Fe releasement to MES medium. b) Amount of Cu and Fe obtained from the ICP-OES analysis of the medium (n = 3). c) Percentage of survived *E. coli* in the medium after 24 h incubation (n = 3). d) Scheme of the ISO 20743 procedure used for measuring the antibacterial capacity of the different types of textiles. As control, textiles with no antibacterial activity were used (n = 3). e) Antibacterial capacity obtained when the ISO 20743 procedure was applied to the different types of textiles.

As shown in Fig. 2b, the first shell fit was performed considering a unique Fe–C/N bond distance and implementing the coordination number as a free parameter. EXAFS spectra of PB-modified textiles after microbiological reduction were similar to those of the starting sample, maintaining clear features associated with the original PB structure. Hence, EXAFS confirmed XANES results indicating that microbiological reduction of textiles containing PB was not altering the PB structure. Since this was happening in both PB-modified and PB/CuO-modified textiles, it may be concluded that both materials were structurally similar, only presenting a slightly different disorder in the second shell. This disorder was confirmed by the much higher Debye-Waller factor in the samples containing both NPs. The changes reported after chemical and microbiological reduction/oxidation processes agreed with those discussed above in the case of textiles without CuO-NPs, confirming that the presence of CuO-NPs did not affect the accessibility and reactivity of PB-NPs on the textile.

### 3.2 NPs stability in the textile: impact on antibacterial activity

The pilot line installed at Klopman produced biocidal fabrics at a feed rate of 0.5 m/min, yielding materials that remained stable for years and retained antibacterial activity over 20 washing cycles (40 min in water at 40 °C under stirring and dried under vacuum overnight) without significant changes in particle concentration [37]. To assess the impact of production speed on NPs stability, a new batch was prepared at an increased feed rate of 10 m/min. The faster coating conditions produced materials with reduced CuO-NPs binding strength and shorter lifespans, losing antibacterial activity after 3–4 washing cycles. This reduced stability enabled the study of bacterial-sensing activity and the influence of NPs co-immobilizations within shorter timeframes.

The stability of CuO and PB-NPs in the smart textile was evaluated using ICP-OES after 24 h of incubation in MES buffer (Fig. 3a). Non-modified cotton-polyester textiles and PB-coated textiles were used as controls.

The stability of the NPs depended on the coating configuration: single-layer coatings (either CuO or PB) exhibited reduced stability compared to dual-layer ones (PB/CuO) (Fig. 3b). Statistical analysis confirmed that iron release from PB/CuO-coated textiles was significantly lower than from PB-coated textiles (P < 0.0001). Specifically, iron release was over tenfold lower in PB/CuO-coated textiles than in PB-only samples (0.67 ± 0.07 mg/L vs. 0.03 ± 0.01 mg/L), suggesting that the initial CuO layer enhanced the stability of the PB coating. This additional stability may arise from either a seeding layer effect, where PB exhibited stronger binding to CuO-NPs than to the hydroxyl groups in the textile, or a redox-based synergistic mechanism, where electron exchange between PB and CuO-NPs enhanced their stability.

Similarly, copper release in PB/CuO-coated samples was also significantly lower than in CuO-coated textiles (P < 0.05). Copper release from PB/CuO-coated textiles was approximately halved compared to CuO-only samples (0.59 ± 0.04 mg/L vs. 0.34 ± 0.09 mg/L), indicating that the PB nano-coating stabilized CuO-NPs, reducing their release to the medium. This effect may also be linked to the previously mentioned redox-based synergistic mechanism.

This stabilizing effect was confirmed through antibacterial activity tests (Fig. 3c). MES buffer solutions from textile incubations were exposed to *E. coli* for 18–24 h to assess bacterial viability. Solutions from CuO-coated textiles presented higher bactericidal activity (survival percentage = 40 %) compared to PB-coated (75 %) and PB/CuO-NPs (75 %) textiles. This higher bactericidal effect was attributed to an increased copper release in the CuO-only samples at the time of the assay.

To further study the influence of the PB coating on antibacterial activity, textile fragments were analysed following the ISO 20743:2021 (Fig. 3d) and classified according to A value (Equation (1) in the Experimental section) into three categories: i) non-antibacterial (A < 1), ii) significantly antibacterial (1 ≤ A < 4), and iii) strongly antibacterial (A ≥ 4). Non-modified and PB-coated textiles presented A values below 1 (Fig. 3e), confirming no significant antibacterial activity. In contrast, CuO-coated and PB/CuO-coated textiles exhibited A values above 4, indicating strong antibacterial activity. Notably, no significant differences were observed between CuO-only and PB/CuO samples, confirming that the PB nano-coating did not compromise the bactericidal properties of the CuO layer.

In summary, the PB coating enhanced the performance of antibacterial textiles by increasing CuO-NPs stability and shelf life, without diminishing the bactericidal activity of the CuO layer.

### 3.3 In situ antibacterial activity assessment: detection of live bacteria through a colour change in the smart textile

**Fig. 4:**
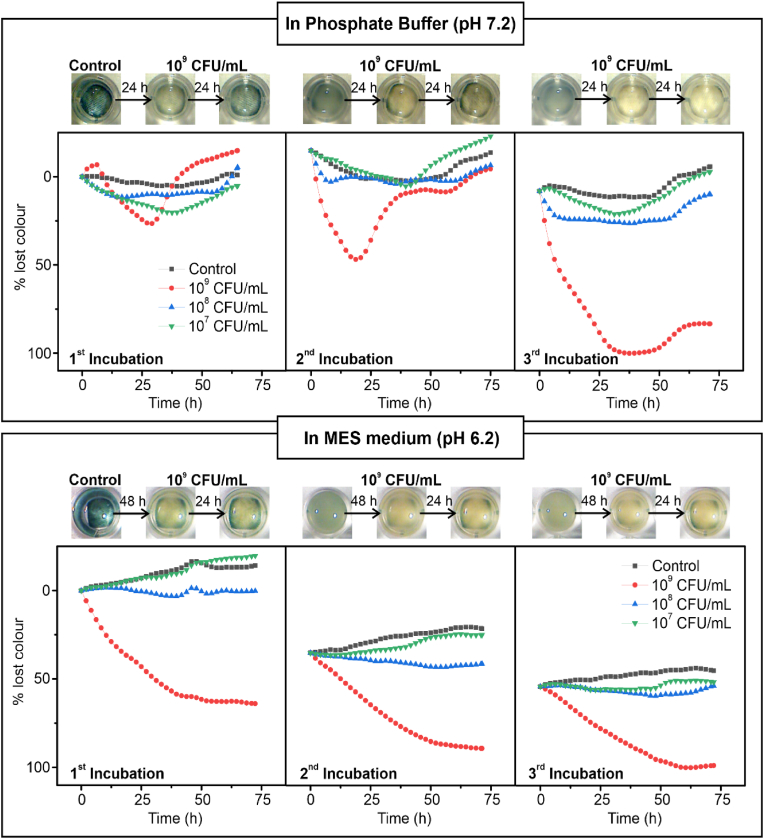
Sensing and antibacterial activities of the smart textiles. Colour lost percentage of polyester-cotton textiles modified with PB- and CuO-NPs after three consecutive incubations of *E. coli* 10^9^, 10^8^ and 10^7^ CFU/mL. The experiment was performed in two different media, phosphate buffer (pH 7.2) and MES medium (pH 6.2) and the images correspond to the smart textiles change of colour upon contact with *E. coli* 10^9^ CFU/mL.

The response of the smart textiles to bacterial colonization was evaluated using *E. coli* suspensions at concentrations of 10^9^, 10^8^ and 10^7^ CFU/mL in two media: phosphate buffer (pH 7.2) and MES medium (pH 6.2). The protocol involved three consecutive 72-h incubations, ensuring a sustained high concentration of viable bacteria in contact with the smart textile for a proper textile colonization. Viable bacteria count in suspension and on the textile were assessed using cell counting and live/dead staining with confocal microscopy, respectively. Additionally, bacterial metabolism-induced textile decolouration was monitored through microscopy imaging. Fig. 4 presents the observed colour changes under each experimental condition, along with representative images of textiles incubated with 10^9^ CFU/mL. Due to the inherent variability in bacterial colonization across samples, results from a single representative experiment are shown in the figure.

**Fig. 5:**
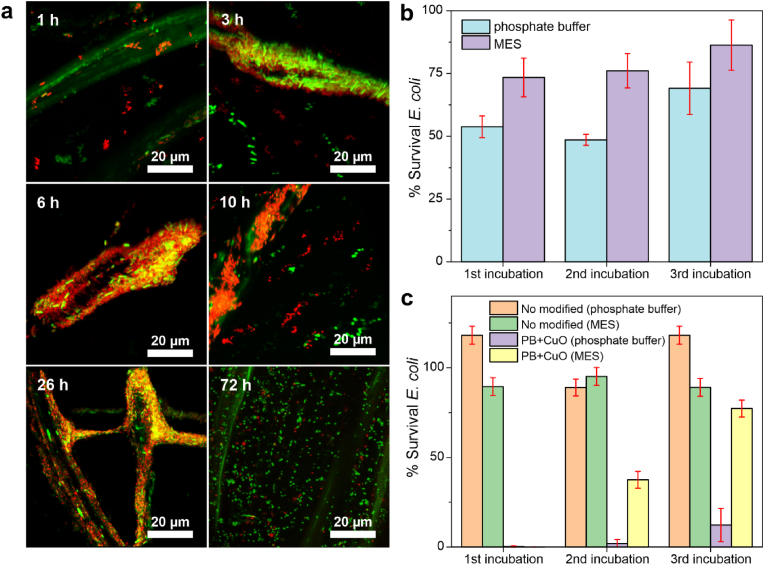
Smart textiles antibacterial mechanisms and live bacteria counts on the textile and in the medium. a) Three-dimensional reconstruction of the confocal images taken at different times (1, 3, 6, 10, 26 and 72 h) of the PB/CuO-modified textile incubated with *E. coli* (10^9^ CFU/mL) in phosphate buffer (pH 7.2) during the first incubation step. b) Survival *E. coli* percentage on the textile fibres obtained from confocal microscopy images (n = 5). c) Survival *E. coli* percentage obtained on the medium (Phosphate buffer and MES) after being in contact with the non-modified textiles and textiles with PB- and CuO-NPs for 72 h during three incubation steps (n = 2).

In phosphate buffer, the first incubation produced minor colour change across all bacterial concentrations. Noticeable colour loss (around 25 %) was only observed for 10^9^ CFU/mL samples after 28 h of incubation, with complete colour recovery (0 % colour loss) by 50 h (see pictures in Fig. 4, top left). This colour recovery was attributed to the combined effects of the textile contact killing mechanism and the re-oxidation of reduced PB by environmental oxygen upon bacterial death. This phenomenon was corroborated through confocal microscopy (Fig. 5a). Confocal microscopy images showed a progressive bacterial attachment to the cotton fibres, with 70–75 % of bacteria remaining viable (green) during the 10 first hours of incubation (Fig. 5b). This viable bacterial population was sufficient to reduce metabolically PB to PW. Over time, bacterial viability decreased by the biocidal activity of CuO-NPs. After 72 h, only 53 % of the initially attached bacteria remained viable, enabling PB re-oxidation to PW by environmental oxygen, and restoring the blue colour of the smart textile. While 47 % of attached bacteria were eliminated through direct contact with the CuO-NPs, copper ion diffusion into the surrounding medium further reduced the survival of suspended bacteria below 1 % (Fig. 5c). These results confirmed the potent antibacterial action of CuO-NPs and highlighted the increased resistance of surface-attached bacteria, which delayed bactericidal kinetics during surface colonization [46].

The second incubation yielded similar results, with survival rates of 49 % on the textile and 1.9 % in suspension. However, bacterial sensing was enhanced, with faster and more intense textile discolouration (50 % colour loss in less than 20 h) and 90 % colour recovery within 40 h. This faster response was attributed to the bacterial surface colonization on the textile, which facilitated bacterial recruitment and surface colonization [47,48].

During the third incubation, the antibacterial performance of the textile declined significantly, with bacterial viability increasing to 69 % on the surface and 12 % in suspension. Colour loss was rapid, with the textile completely losing its colour in 30 h. Unlike earlier incubations, colour recovery did not occur, likely due to the high number of live bacteria attached to the textile. PB reduction was observed for the first time in 10^8^ CFU/mL sample, confirming a significant antibacterial activity loss after three incubation cycles.

The response of the smart textile was also studied in MES medium for comparison. The MES medium (pH 6.2), being more acidic than the phosphate buffer (pH 7.2), is expected to: (i) enhance the stability of PB-NPs within the textile [49], (ii) promote the release of CuO-NPs into the medium [50], and (iii) create a less physiological environment for bacteria [51]. However, previous studies demonstrated that *E. coli* proliferation in phosphate buffer and MES (both supplemented with glucose) does not differ significantly [23].

Experimentally, the three 72-h incubation trials in MES exhibited consistent colorimetric changes, with a gradual decolouration of the textile due to bacterial reduction, stabilizing after approximately 50 h of incubation (Fig. 4). Unlike the phosphate buffer experiments, the textile did not recover its original colour during the incubation period, a result attributed to the high concentrations of live bacteria colonizing the textile.

Confocal microscopy confirmed this observation, showing bacterial survival rates on the textile of 73 %, 76 %, and 86 % for the first, second, and third incubation steps, respectively (Fig. 5b). This progressive reduction in contact-killing efficiency was linked to the lower stability of CuO-NPs in the MES medium and their rapid release into the surrounding solution. Indeed, the proportion of live bacteria in the medium (Fig. 5c) increased markedly, from nearly negligible levels (0.03 %) during the first incubation, to 37.5 % and 77 % during the second and third incubation steps, respectively. The decline in the antibacterial efficacy of diffused CuO-NPs suggested that the majority of these particles were released during the first incubation step, leaving only minimal amounts retained within the textile. As a result, the smart textile experienced a progressive reduction in its biocidal activity in MES medium, ultimately leading to pronounced colour changes. These changes visually indicated the presence of bacteria, aligning with the expected sensing mechanism, after a significant reduction in antibacterial action.

These findings indicate that the smart biocidal textile reliably responds to the presence of live bacteria through a distinct, observable colour change. This response serves as an indicator of bacterial colonization and the potential presence of infective bacteria. Furthermore, the colour change provides an in-situ assessment of the textile loss of biocidal capacity, facilitating timely identification of its diminished antibacterial efficacy independently on the experimental conditions.

### 3.4 Impact of smart textiles on skin: cytotoxicity and on-textile proliferation studies

Smart textiles are designed for direct contact with the skin, making crucial to evaluate their impact on cell viability and proliferation. The cytotoxicity of PB and PB/CuO-coated textiles was assessed *in vitro* using HDF-a as cellular model of human skin. For comparison, non-modified poly-cotton and CuO-coated textiles were also included in the analysis.

The cytotoxicity of the materials was determined using the MTT assay, where dermal cells were exposed to increasing amounts of the textile samples for 24 h. Following exposure, textile fragments were removed, and the cells were incubated with the MTT reagent. Cellular metabolism converted the reagent into purple formazan, the concentration of which was proportional to the number of viable cells. To minimize inter-assay variability, cell viability was expressed as a percentage relative to control wells containing untreated cells.

**Fig. 6:**
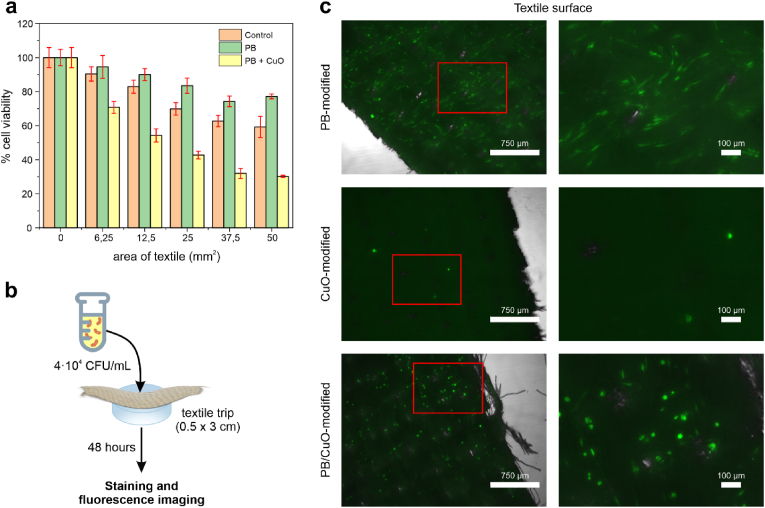
a) MTT cytotoxicity assay obtained for different textile areas (from 6 to 50 mm^2^) for textiles coated with PB and PB/CuO. Cell viability was obtained after 24 h of incubation (n = 3) and viability was expressed as percentage versus non-treated control samples. b) Scheme of the procedure followed to take the fluorescence images to study the proliferation on the textile surface. c) Fluorescence images of the different types of textiles (0.5 × 3 cm) after 48 h of incubation with HDF-a cells. The green points represent live bacteria on the textile samples.

The results for non-modified, PB-coated and PB/CuO-coated textiles are presented in Fig. 6a. A decrease in cell viability was observed for all samples, in the extent of cytotoxicity increasing proportionally to the textile area in contact with cells. However, the level of cytotoxicity varied among samples. Non-modified and PB-coated textiles exhibited higher cell viability compared to PB/CuO-coated textiles, which were consistently more cytotoxic (between 20 and 56 % more cytotoxic, depending on the area of the textile).

The mild cytotoxic effect of non-modified and PB-coated textiles was attributed to a combination of mechanical damage caused by the textile roughness and the potential presence of residual toxic agents in the untreated fabric. This hypothesis was supported by two observations. First, PB-coated textiles exhibited lower cytotoxicity than non-modified textiles, suggesting that the PB coating mitigated mechanical damage and reduce the impact of residual agents initially present in the textile. Second, prior studies have demonstrated that PB-NPs, at the concentration used, are not cytotoxic [23]. In contrast, the greater cytotoxicity observed in PB/CuO-coated textiles was attributed to the additional presence of CuO-NPs, which are known to induce oxidative stress and DNA damage, thereby reducing dermal cell viability [52].

To mimic real-life exposure, a second assay was conducted to assess the proliferation of dermal cells on the textile surface. In this experiment, HDF-a cells were seeded directly onto textile fragments immobilized at the bottom of culture wells and allowed to proliferate for 48 h. The textiles were then removed, stained with CellTracker™ Green CMFDA, and visualized by confocal microscopy (Fig. 6b). CellTracker™ Green CMFDA was selected due to its minimal cytotoxicity, high cell retention, and stable fluorescence, which results from intracellular conjugate formation that prevents leakage. These properties ensured long-term fluorescence stability, making it optimal for proliferation and cytotoxicity studies.

To minimize the effect of diffused toxic agents, a higher medium volume (500 μL *vs.* 150 μL used in the MTT assay) was employed. Imaging of the well bottom confirmed similar cell viability across all samples, indicating that soluble toxic agents did not significantly affect the assay (Supporting Information, Figure S5.1). Regarding on-textile proliferation (Fig. 6c), PB-coated and PB/CuO-coated textiles supported significant cell growth, whereas CuO-coated textiles exhibited reduced proliferation, suggesting pronounced contact cytotoxicity.

These results highlight the excellent performance of the PB coatings, which not only confer bacterial sensing capabilities to the textile, but they also enhance the stability of bactericidal CuO-NPs while mitigating their contact cytotoxicity. Notably, this stabilization was achieved without compromising the antibacterial activity of the textile. The smart textile exhibits comparable sensitivity and response times to state-of-the-art materials, aligning with bacterial loads typically found on highly contaminated surfaces (>10^4^–10^6^ bacteria/cm^2^) [53]. However, it surpasses existing solutions in sensor stability, cost-efficiency, and scalability of the production technology, as summarized in Table 1.

**Table 1:** State of the art of the different smart materials reported with sensing or sensing and antibacterial activities and their main features.

Type of Smart material	Sensing or sensing and antibacterial activities	Sensing mechanism	Response time	Limit of detection	Stability	Selectivity	Cytotoxicity	Scalability	Ref.
Hydrogel wound dressing	Sensing	Phospholipid membrane	6 h	Biofilm formation	Poor	*E. coli, P. aeruginosa, S. aureus and E. faecalis*	No	+	[17]
Hydrogel film coating	Sensing	Doped hydrogel with specific enzymes	1 h	Enzyme concentration: 10^−9^ M	Enzymes have poor stability and low reproducibility	Gram-positive and Gram-negative bacteria	Not reported	+	[54]
Smart textile	Sensing	pH-sensitive (organic dye)	24 h (Gram-positive) and 6 h (Gram-negative)	Not reported	Poor (sensible to pH and temperature changes)	Gram-positive and Gram-negative bacteria	No	+++	[18]
Smart textile	Sensing	pH-sensitive (organic dye)	18 h (Gram-positive) and 12 h (Gram-negative)	Not reported	Poor (sensible to pH and temperature changes)	*E. coli* and *L. acidophilus*	No	+++	[19]
Smart self-assembled multilayer film	Both	pH-sensitive	24 h	Approx. 10^9^ CFU/mL	Poor (sensible to pH and temperature changes)	*E. coli* and *S. aureus*	Not reported	+	[55]
Smart textile	Both	pH-sensitive (organic dye)	24 h	10^8^ CFU/mL	High	*E. coli* and *S. aureus*	No	++	[21]
Smart wearable textile	Both	Oxygen-sensitive compound	18 h	10^6^ CFU/mL	Poor (also sensitive to the presence of transition metals)	*E. coli* and *S. aureus*	Slightly cytotoxic	++	[22]
Multilayer wound dressing	Both	Oxygen-sensitive compound	6 h (without antibacterial agent) and more than 48 h (with antibacterial agent)	O.D = 0.8 (Between 10^8^ and 10^9^ CFU/mL)	High	Gram-positive and Gram-negative bacteria	Yes	++	[20]
Smart wound dressing	Both	pH-sensitive (FRET)	24 h	10^4^ CFU/mL	Poor (sensible to pH changes)	*S. aureus*	No (for short times)	++	[56]
Smart biocide fabric	Both	Metabolic indicator	6 h (without antibacterial agent) and more than 24 h (with antibacterial agent)	Between 10^7^ and 10^9^ CFU/mL	High	Gram-positive and Gram-negative bacteria	No	+++	This work

## 4 Conclusions

Smart textiles with both bactericidal and bacterial-sensing capabilities have been developed to address the risks of bacterial infections associated with contaminated hospital fabrics and wound dressings. Polyester-cotton textiles were successfully coated with CuO and PB nanoparticles through a sonochemical process, achieving a homogeneous nanoparticulate distribution that covered more than 90 % of the textile surface. Despite minor alterations in the iron coordination and partial reduction of iron ions in PB during the coating process, the embedded PB-NPs retained their electrochromic properties and bacterial sensing capacity. The metabolic activity of live bacteria induced a localized discolouration of the textile due to the reduction of PB to uncoloured PW. This colour change was reversible upon bacterial death, with environmental oxygen re-oxidation of PB to restore the original blue colour.

The PB nano-coating significantly enhanced the performance of CuO-coated textiles by doubling the stability of CuO-NPs, and reducing cytotoxicity, while maintaining their antibacterial efficacy. Further, sonochemical coatings did not affect the mechanical properties of the textiles. Additionally, the embedded PB-NPs served as an efficient bacterial-sensing probe, providing a visible, intense colour change and offering a simple method for in-situ bacterial detection when antibacterial properties begin to degrade. This feature offers a straightforward method for in-situ and real-time bacterial detection and potential infection risk assessment.

While further studies are required to optimize the sensitivity, response time and functionality in real-world conditions (e.g., under dry environments), this proof-of-concept demonstrates the potential of these smart textiles as a valuable tool for infection control and prevention in high-risk settings such as hospitals, schools or healthcare centres.

## CRediT authorship contribution statement

**Amparo Ferrer-Vilanova:** Writing – original draft, Visualization, Investigation, Formal analysis, Data curation, Conceptualization. **Josune Jimenez Ezenarro:** Resources, Methodology. **Kristina Ivanova:** Writing – review & editing, Validation, Resources. **Óscar Calvo:** Investigation. **Ilana Perelshtein:** Writing – review & editing, Investigation. **Giulio Gorni:** Writing – review & editing, Resources, Methodology, Investigation. **Ana Cristina Reguera:** Writing – review & editing, Investigation. **Rosalía Rodríguez-Rodríguez:** Writing – review & editing, Resources, Investigation. **Maria Blanes:** Investigation. **Núria Vigués:** Validation, Resources. **Jordi Mas:** Validation, Resources. **Aharon Gedanken:** Writing – review & editing, Investigation. **Tzanko Tzanov:** Writing – review & editing, Validation, Project administration, Conceptualization. **Gonzalo Guirado:** Writing – review & editing, Methodology, Conceptualization. **Xavier Muñoz-Berbel:** Writing – review & editing, Writing – original draft, Supervision, Methodology, Funding acquisition, Conceptualization.

## Declaration of competing interest

The authors declare that they have no known competing financial interests or personal relationships that could have appeared to influence the work reported in this paper.

## Data Availability

Data will be made available on request.
