# Supplementary material for: Smart bactericidal textile enabling in-situ visual assessment of antimicrobial activity
Source: Mater Today Bio. 2025 Apr 3;32:101724. doi: 10.1016/j.mtbio.2025.101724 (PMC12000729; doi:10.1016/j.mtbio.2025.101724)
Supplement: Multimedia component 1 [file mmc1.docx]

Supporting Information

**Smart Bactericidal Textile Enabling In-Situ Visual Assessment of Antimicrobial Activity**

**Amparo Ferrer-Vilanova^1*^, Josune Jimenez Ezenarro^1^, Kristina Ivanova^2^, Óscar Calvo^3^, Ilana Perelshtein^4^, Giulio Gorni^5,6^, Ana Cristina Reguera^7^, Rosalía Rodríguez-Rodríguez^7,8^, Maria Blanes^3^, Núria Vigués^9^, Jordi Mas^9^, Aharon Gedanken^4^, Tzanko Tzanov^2^, Gonzalo Guirado^10^, Xavier Muñoz-Berbel^1,11^***

^1^Institut de Microelectrònica de Barcelona (IMB-CNM, CSIC), Universitat Autònoma de Barcelona, 08193, Cerdanyola del Vallès (Barcelona), Spain.

^2^Universitat Politècnica de Catalunya, Edifici Gaia, Pg. Ernest Lluch/Rambla Sant Nebridi s/n. 08222, Terrassa (Barcelona), Spain.

^3^Asociación de Investigación de la Industria Textil – AITEX, Área de I+D, Grupo de Investigación en Eco-procesos, Cosmética y Salud. Plaza Emilio Sala, 1, 03801 Alcoi (Alacant), Spain.

^4^Department of Chemistry, and the BINA center, Bar-Ilan University, 5290002 (Ramat-Gan), Israel.

^5^CELLS-ALBA Synchrotron, Carrer de la Llum 2-26, 08290 Cerdanyola del Vallès (Barcelona), Spain

^6^Instituto de Óptica (IO-CSIC), c/Serrano 121, 28006 Madrid, Spain.

^7^Basic Sciences Department, Faculty of Medicine and Health Sciences, Universitat Internacional de Catalunya (UIC), Sant Cugat del Vallès, E-08195, Spain.

^8^Centro de Investigación Biomédica en Red de Fisiopatología de la Obesidad y la Nutrición (CIBEROBN), Instituto de Salud Carlos III, Madrid, E-28029, Spain

^9^Departament de Genètica i Microbiologia, Universitat Autonòma de Barcelona, 08193, Cerdanyola del Vallès (Barcelona), Spain.

^10^Departament de Química, Universitat Autonòma de Barcelona, 08193, Cerdanyola del Vallès (Barcelona), Spain.

^11^CIBER de Bioingeniería, Biomateriales y Nanomedicina, Instituto de Salud Carlos III, Madrid, E-28029, Spain

**S1.** **Dynamic Light Scattering Results**

Dynamic Light Scattering (DLS) was employed to determine the hydrodynamic diameter and polydispersity index (PDI) of the Prussian Blue nanoparticles (PB-NPs) in dispersion. The PDI is a dimensionless parameter that quantifies the width of the particle size distribution in a sample, ranging from 0 (monodisperse) to >1 (highly polydisperse). Its magnitude depends on the variance of the particle size distribution (σ), and the Z-average hydrodynamic diameter (D) according to the following expression:

$$PDI=\frac{\sigma^{2}}{D^{2}}$$

DLS was measured two times in independent samples. Representative results are illustrated in Figure S1:

Figure S1. Representation of the hydrodinamic particle size distribution obtained by DLS.

The Z-average hydrodynamic diameter of the PB-NPs was found to be 90.35 nm in one dataset and 76.54 nm in another, indicating some variation in size measurements. The PDI values were 7.18 and 6.53, respectively, suggesting a highly polydisperse system with potential aggregation effects.

**S2. Mechanical Properties**

The main mechanical properties of the fabrics coated with CuO-NPs by Klopman were investigated to study the influence of the sonochemical coating process. This study was realized by implementing different international standard methods descrived in the Table S2.1.

**Table S2.1.** International standard methods used for the study of the mechanical properties of the textiles coated with CuO-NPs by the sonochemical coating process and their description.

| **Standard** | **Short description of the testing method and its scope (as described in the standard)** |
| --- | --- |
| ISO 13934-1:2013. Textiles — Tensile properties of fabrics — Part 1: Determination of maximum force and elongation at maximum force using the strip method | ISO 13934-1:2013 specifies a procedure to determine the maximum force and elongation at maximum force of textile fabrics using a strip method. |
| ISO 12947-2:2016. Textiles — Determination of the abrasion resistance of fabrics by the Martindale method — Part 2: Determination of specimen breakdown | ISO 12947-2:2016 specifies the procedure for the determination of specimen breakdown (end-point of test) by inspection at fixed intervals and is applicable to all textile fabrics including nonwovens apart from fabrics where the specifier indicates the end performance as having a low abrasion wear life. |
| ISO 12945-2:2000. Textiles — Determination of fabric propensity to surface fuzzing and to pilling — Part 2: Modified Martindale method | This part of ISO 12945 specifies a method for determination of the resistance to pilling and surface change of textile fabrics using a modified Martindale method. |

Below, Table S2.2 shows the results obtained with the Klopman fabric samples coated with the pilot line, using Cu(Ac)_2_ and a speed range from 0.5 to 10 m/min. No significant changes were observed on the pilling and abrasion resistance, in a comparison with the uncoated fabric.

**Table S2.2**. Results obtained with the Klopman fabric samples coated with the pilot line, using Cu(Ac)_2_ and a speed range from 0.5 to 10 m/min.


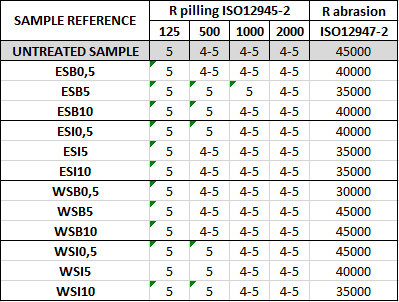


Analogously, results for Klopman fabric samples coated with the pre-commercial line, using 1 g/L of Cu(Ac)_2_ and being processed at 5 m/min are shown in Table S2.3. No significant changes were observed on the mechanical properties, being compared with the uncoated fabric.

**Table S2.3**. Results obtained with the Klopman fabric samples coated with the pre-commercial line, using 1 g/L of Cu(Ac)_2_ and being processed at 5 m/min.


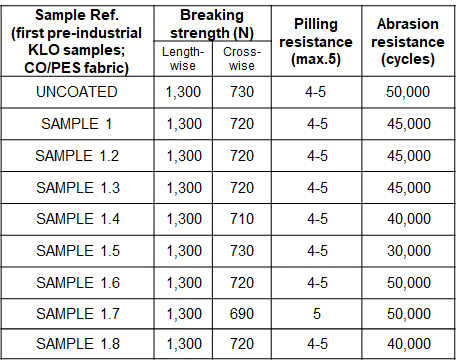


Tables previously shown prove that it was not detected any significant variation among treated and untreated fabrics. Therefore, the sonochemical coating procedure does not affect the mechanical quality of the fabric in a negative way.

**S3. EDX Measurements**

**
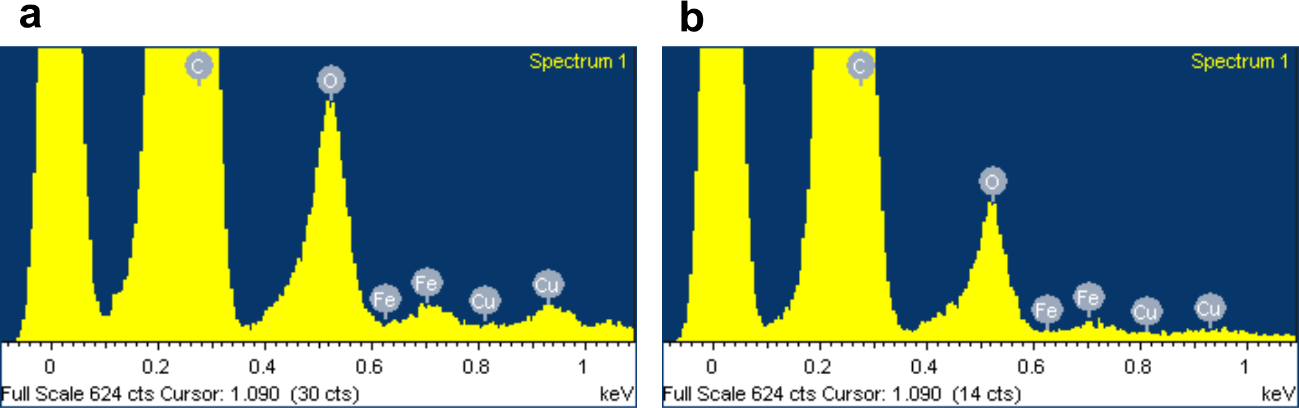
**

**Figure S3.1.** a) EDX spectrum of the textiles modified with PB- and CuO-NPs after reduction with NaBH_4_ (10kV). b) EDX spectrum of the textiles modified with PB- and CuO-NPs after reduction with NaBH_4_ and re-oxidation with H_2_O_2_ (10 kV).

**S4. XANES and EXAFS measurements**

**Table S4.1.** Results of the EXAFS fit. Deg is the degeneracy of the scattering path (that corresponds to the coordination number for single scattering paths), R is the bond distance and σ^2^ the Debye-Waller factor. The many body amplitude reduction factor S_0_^2^ was obtained from fitting the Fe foil and fixed to 0.8.

| **Sample** | **Shell** | **N/Deg** | **R (Å)** | **σ^2^ (10^-3^ Å^2^)** |
| --- | --- | --- | --- | --- |
| ***PB*** |  | | | |
| Starting compound | Fe-C | 3 | 1.93(2) | 6(2) |
|  | Fe-N | 3 | 2.04(2) | 6(2) |
|  | Fe-C-N | 12 | 3.14(3) | 8(1) |
| Red. Bac | Fe-C | 3 | 1.92(2) | 7(3) |
|  | Fe-N | 3 | 2.11(3) | 7(3) |
|  | Fe-C-N | 12 | 3.13(3) | 10(2) |
| ***PB+CuO*** |  | | |  |
| Starting compound | Fe-C | 3 | 1.93(2) | 7(2) |
|  | Fe-N | 3 | 2.10(4) | 7(2) |
|  | Fe-C-N | 12 | 3.13(3) | 17(2) |
| Red. Bac | Fe-C | 3 | 1.90(2) | 7(4) |
|  | Fe-N | 3 | 2.05(3) | 7(4) |
|  | Fe-C-N | 12 | 3.13(3) | 17(2) |

**S5. Cytotoxicity Assays**


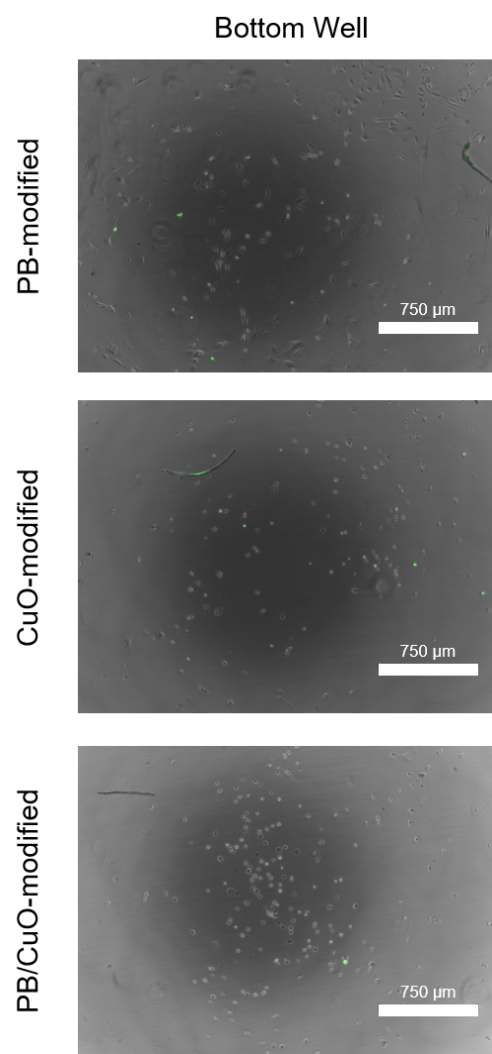


**Figure S5.1.** Confocal microscopy images of the bottom of the well of the different textiles studied. In all cases, a similar number of viable cells is observed, confirming the low influence of diffused toxic agents on the assay.
